# A retrospective study of environmental predictors of dengue in Delhi from 2015 to 2018 using the generalized linear model

**DOI:** 10.1038/s41598-022-12164-x

**Published:** 2022-05-16

**Authors:** Poornima Suryanath Singh, Himanshu K. Chaturvedi

**Affiliations:** 1 University School of Medicine and Paramedical Health Sciences, Guru Gobind Singh Indraprastha University, New Delhi, 110075 India; 2 ICMR-National Institute of Medical Statistics, Indian Council of Medical Research, Ansari Nagar, New Delhi, 110 029 India

**Keywords:** Environmental social sciences, Diseases, Health care

## Abstract

Dengue fever is a mosquito-borne infection with a rising trend, expected to increase further with the rise in global temperature. The study aimed to use the environmental and dengue data 2015–2018 to examine the seasonal variation and establish a probabilistic model of environmental predictors of dengue using the generalized linear model (GLM). In Delhi, dengue cases started emerging in the monsoon season, peaked in the post-monsoon, and thereafter, declined in early winter. The annual trend of dengue cases declined, but the seasonal pattern remained alike (2015–18). The Spearman correlation coefficient of dengue was significantly high with the maximum and minimum temperature at 2 months lag, but it was negatively correlated with the difference of average minimum and maximum temperature at lag 1 and 2. The GLM estimated β coefficients of environmental predictors such as temperature difference, cumulative rainfall, relative humidity and maximum temperature were significant (*p* < 0.01) at different lag (0 to 2), and maximum temperature at lag 2 was having the highest effect (IRR 1.198). The increasing temperature of two previous months and cumulative rainfall are the best predictors of dengue incidence. The vector control should be implemented at least 2 months ahead of disease transmission (August–November).

## Introduction

Dengue fever, impacting half the world’s population mainly the Tropical and Sub Tropical regions of the world, is an arboviral illness caused by Dengue viruses (DENV-1, DENV-2, DENV-3, and DENV-4), which are transmitted by the bite of infected female Aedes mosquito (*Aedes aegypti* and *Aedes albopictus*)^1^. Dengue disease severity contrasts ranging from a mild flu-like syndrome known as dengue fever (DF) to more severe forms of the disease such as dengue haemorrhagic fever (DHF), and dengue shock syndrome (DSS)^2^. Currently, there is no antiviral drug for the treatment of dengue fever, the vaccine against all serotypes of the dengue virus is also not available and the treatment is limited to symptomatic patient management regimens.

More than 128 countries across all regions of WHO are endemic to Dengue with Western Pacific regions and South-East Asia being more affected^3^. WHO has reported that in the last 50 years there has been a geographic expansion of dengue to new places thus the number of countries being affected has increased by 30-fold and the expansion has been from urban to rural settings^4^.

In India, dengue fever is a vital public health problem in most of the states. In the early twentieth century, dengue was endemic in a few northern states (Haryana, Punjab, Delhi, Rajasthan and Chandigarh) and southern (Maharashtra, Tamil Nadu, Puducherry and Karnataka). However, in recent years, it has been spread to many other States and Union Territories^5^. Dengue outbreak in Delhi has been a serious public health issue as it has been regularly reported during different years; ever since major outbreaks in 1996, and thereafter, in 2003, 2006, 2010, 2013 and 2015^3,6^.

Several studies were conducted worldwide to demonstrate that climatic factors, such as temperature, rainfall, and humidity are the most important correlates of dengue transmission and changes in these factors act as determinants of the intensity and period of transmission^1,7–10^. This could be accounted for by the indirect effect of climatic factors on the incidence of dengue through their influence on the lifecycle of both the vector and virus^9^. Global temperatures are projected to rise by 2 °C or more by the end of the twenty-first century^11^. Relationships between dengue incidence and El Niño episodes are shown in multiple studies, indicating climate and weather can affect the *Aedes* mosquitoes and DENV through multiple/interrelated mechanisms^1^. According to the Intergovernmental Panel of Climate Change (IPCC) up until 2080, there may be 3.5 billion people worldwide who have to face the risk of DF infection due to climate change and the effects of global warming^12^.

While there have been many published works investigating the relationship between climatic variables and dengue, the relationship between climatic factors and dengue incidence varies with countries and geographical locations^1,13^. There is also a need to re-examine the modified association between climatic variables and dengue over time due to climate change. In the absence of appropriate therapy to treat dengue and the still ongoing vaccine development efforts, only education and vector control remain central for dengue prevention and control. Adding on to the situation, many endemic countries where dengue is likely to spread further have underdeveloped health systems, increasing the considerable challenges of disease prevention and control. The identification of factors, particularly environmental variables that can be used to predict epidemics is imperative to allow sufficient time for health systems to be prepared. This will further improve our understanding of how a changing climate may contribute to the geographic extension of mosquitoes and disease into new areas^1^.

The change in climatic conditions makes it imperative to revisit and re-examine the issue of changing climate with the occurrence of dengue cases. The present study aimed to assess the seasonal variation in the occurrence of dengue in Delhi, and its environmental correlates for the period from 2015 to 2018. The impact of environmental factors as predictors of dengue was also an important part of the study.

## Methods

The National Capital Territory (NCT) of Delhi covering an area of 1484 km^2^, and a population of 16,753,235 with a density of 11,297 persons per km^2^ is one of the largest cities in the country. The city has recorded high population growth in the last two decades which was mainly due to the migration of people and caused by increasing urbanization.

### Data source

As dengue is a notifiable disease, all laboratory diagnosed positive cases were reported and recorded by the health administration of Delhi. Computerised data of dengue cases were obtained from January 2015 to December 2018 (4 years) from the Municipal Corporation of Delhi, a government administration maintaining the data. The seroepidemiological data provide information of locally-acquired dengue confirmed cases in Microsoft Excel format with age, sex, locality of residence, zone, name of the hospital, date of admission, date of notification, date of discharge, etc. The data required for the study was extracted from the main database and only clinically confirmed positive cases with other information were included in the analysis. The meteorological data of Delhi were gathered from Indian Meteorological Department, Pune which includes monthly averages of maximum temperature (°C), minimum temperature (°C), total rainfall (mm), and relative humidity (%) for the study period from January 2015 to December 2018.

### Data analysis

The environmental variables such as total rainfall, cumulative rainfall, maximum and minimum temperatures and humidity including the monthly incidence of dengue cases were included in the analysis to know the seasonal distribution and its correlation. The lagged period of 1 to 3 months of the environment variables were used to know the previous month's relation with the current dengue case including no lag (zero month lag). The three-month lag length was considered for analysis as it was sufficient to cover development from the egg, extrinsic and intrinsic incubation period of the virus to the patient's hospital visit following the onset of symptom^13^. Due to the non-linear relationship between dengue cases and climate factors, the Spearman correlation was computed to identify the most influencing environmental factors including the preceding months (lag period) on the occurrence of dengue (*p* < 0.05).

Dengue cases were count variables, Poisson or Negative Binomial was the possible probability distribution to choose. The variance (88694.05) in the seasonal data of dengue was much higher than the mean of dengue cases (98.31), which shows that dengue cases were over-dispersed. Hence, Generalized Linear Model (GLM) using Negative Binomial Regression (NBR) for dengue cases as a response and environment variables as predictors were used for modelling. Various models were attempted with several combinations of environmental variables (all or subsets) and finally, the best fit model was selected for the prediction of dengue compared with the likelihood ratio, mean deviance and AIC (Akaike's Information Criterion) values of models (*p* < 0.05). The Incidence Rate Ratio (IRR) was calculated to know the relative risk of dengue incidence with climatic factors. The statistical analysis of the data was performed using IBM SPSS software (ver. 23).

### Ethics approval

The study received approval from the School Research Committee of the university and also ethical approval from the Ethics Committee of the Institute constituted by the Indian Council of Medical Research, New Delhi. The environmental data used in this study was provided by the Indian Meteorological Department, Govt, of India and data on dengue cases for the year 2015–2018 was provided by the Municipal Corporation of Delhi, Govt. of Delhi after getting approval of the study. The study used the secondary data of dengue cases available with the Municipal Corporation of Delhi, so the patient's consent was not required. All research activities were carried out in accordance with guidelines and regulations of the Indian Council of Medical Research (ICMR).

### Consent to participate

The informed consent was waived by the institutional ethics committee of the Indian Council of Medical Research.

## Results

Total 4179 confirmed dengue cases were recorded in four years, the highest number of cases (3189) was recorded in 2015, but it was declined in 2016 (545 cases), again slightly increased in 2017 (706 cases), and there was a sharp decline in 2018 (279 cases). Descriptive meteorological statistics are presented in Table 1, for the study period 2015–18 revealed that the average maximum temperature ranged between 17.9 and 40.9 °C, whereas the average minimum temperature ranged between 6.7 and 29.6 °C. The average relative humidity ranged from 23.1 to 94.5% with mean humidity of 53.52%. The highest total rainfall in the study period was 295 mm with a mean rainfall 58.98 mm. The difference between the monthly minimum and maximum temperature showed a range between 7 and 16.8. The minimum difference was in the year 2018 and the maximum difference was in the year 2016.

**Table 1:** Descriptive analysis of environmental data of Delhi, 2015–18 (48 months).

Variable	Mean	Min	Max	SD
Average max temperature (°C)	30.02	17.9	40.9	6.05
Average min temperature (°C)	19.5	6.7	29.6	7.37
Average temperature(°C)	25.76	13	34.8	6.58
Difference min–max temperature (°C)	12.51	7	16.8	2.94
Average relative humidity (%)	53.52	23.1	94.5	15.08
Total rainfall (mm)	58.983	0	292.5	84.65
Cumulative rainfall (mm)	350.35	0	824.6	305.58

*SD* standard deviation.

**Figure 1:**
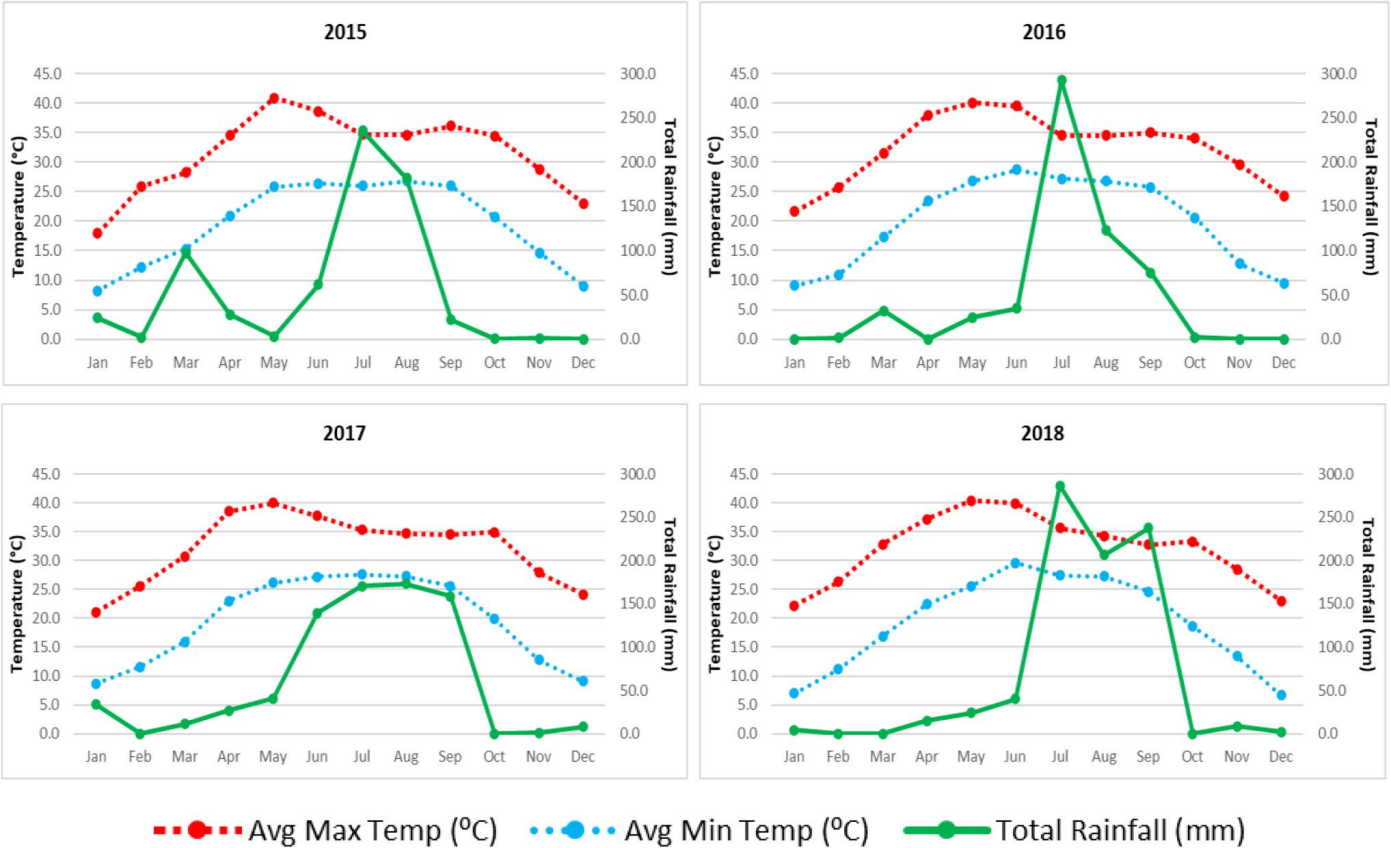
Line Graph showing the month-wise pattern of environmental variables in the year 2015–2018 in Delhi.

Figure 1 depicts the monthly variations in mean maximum temperature, mean minimum temperature, total rainfall and average relative humidity. The temperature parameters seem to relate closely to rainfall and humidity in each month. The monthly rainfall variations indicate that the monsoon season extends from June to September, thereby raising the probability of dengue fever occurrence from < 1% in June to > 28% in September.

**Figure 2:**
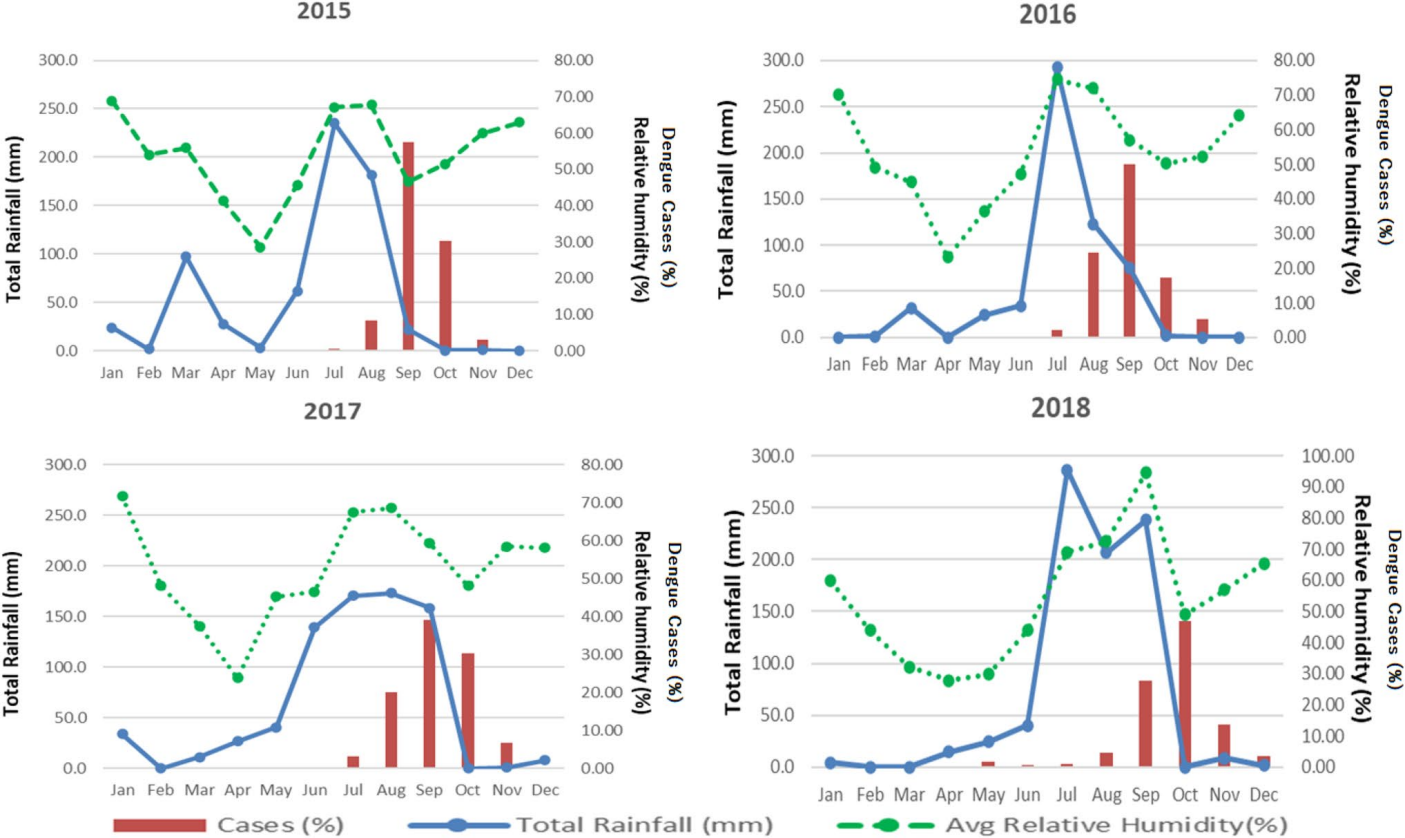
Monthly occurrences of dengue cases and average total rainfall with humidity, 2015–18, Delhi, India.

The seasonal pattern of occurrence of dengue cases revealed that the cases were raised in the monsoon and post-monsoon seasons. Dengue cases or its monthly proportion were highest from August to October across the years 2015 to 2018. The distribution of dengue cases with humidity revealed that the peak of reported cases was recorded when there was a fall in the humidity and this was a consistent pattern in the past four years (Fig. 2). The range of humidity was from 49 to 60% during the peak month of dengue. A seasonal pattern of dengue occurrence was observed with cases that progressed from July to August, hit the highest point in September to October and declined by December. The highest dengue incidence shifted from September outbreak to October in 2017 and 2018. Rainfall and humidity showed an upward trend beginning in May, while the mean minimum temperature and the mean maximum temperatures drop. This appears to be closely linked with the rise in dengue cases in July every year.

### Seasonal pattern of Dengue

**Figure 3:**
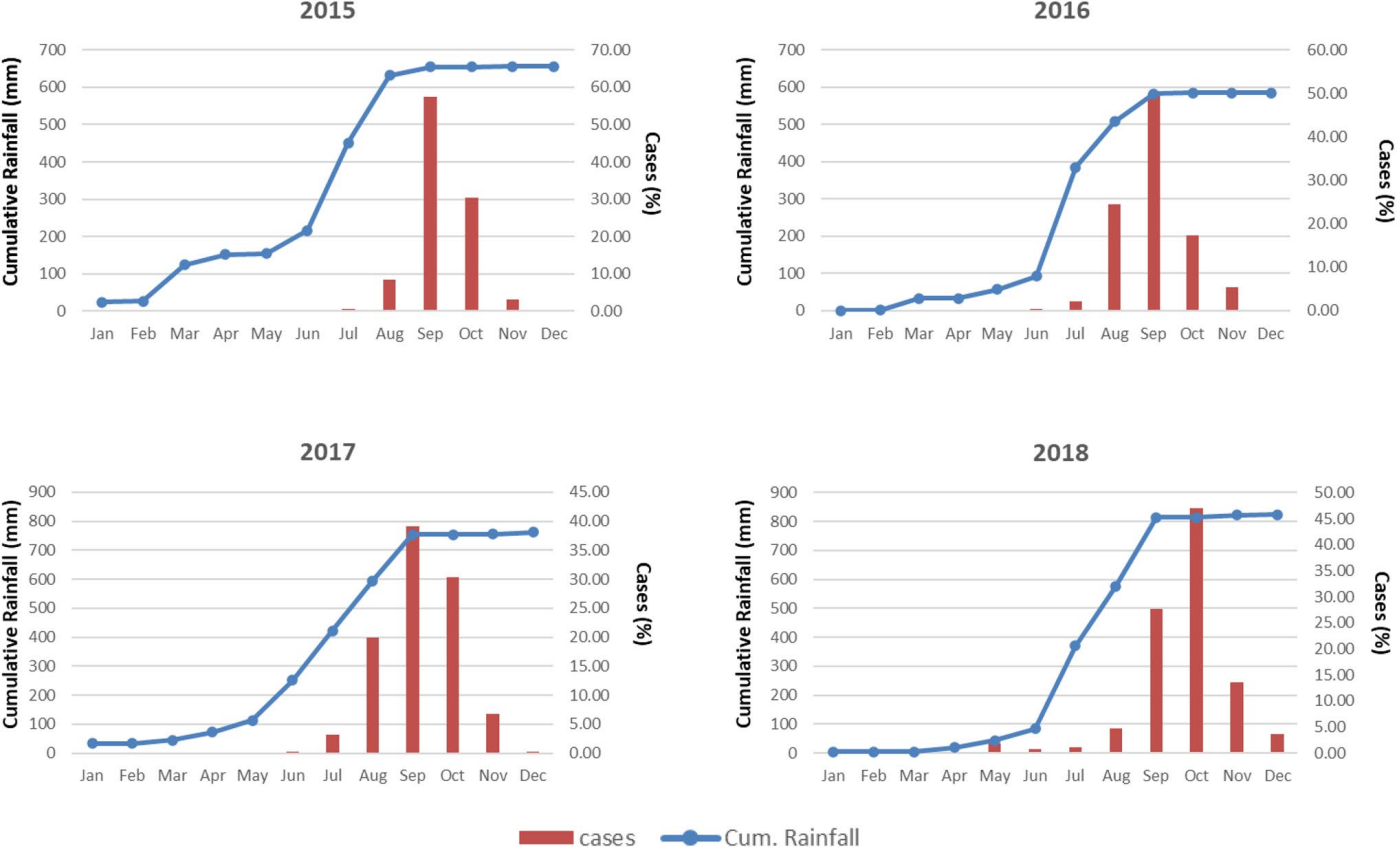
Monthly occurrences of dengue cases and cumulative rainfall, 2015–18, Delhi, India.

Seasonal analysis of dengue cases with cumulative rainfall revealed that the number of reported cases was highest in September and October across the study period (2015 to 2018) when the cumulative rainfall was also recorded high (Fig. 3). During the peak of dengue, the cumulative rainfall ranged from 590 to 810 mm.

**Figure 4:**
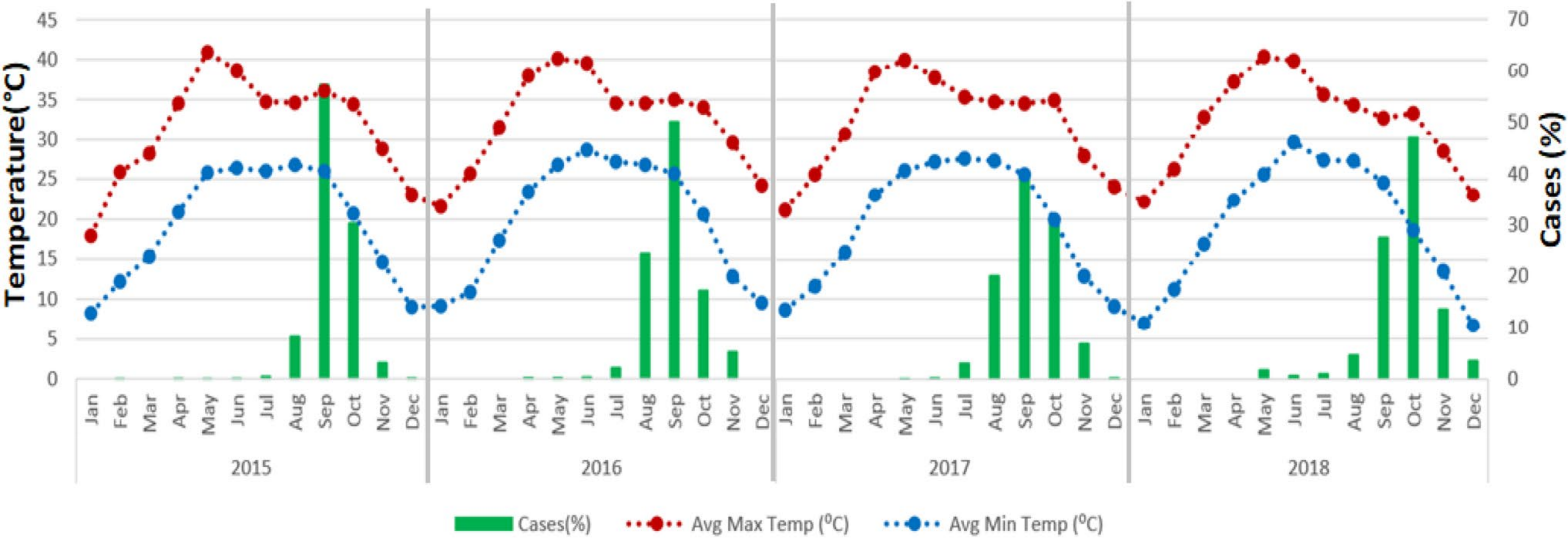
Monthly occurrences of dengue cases and temperature, 2015–18, Delhi, India.

The seasonal pattern of dengue cases with average maximum and minimum temperature revealed that the number of reported cases was the highest from August to October across the study period (2015 to 2018) which was a few months after the highest recorded maximum and minimum temperature. The dengue cases reached their peak following the months with the highest temperature (Fig. 4). During the peak of dengue, the temperature ranged from 24 to 35 °C.

**Figure 5:**
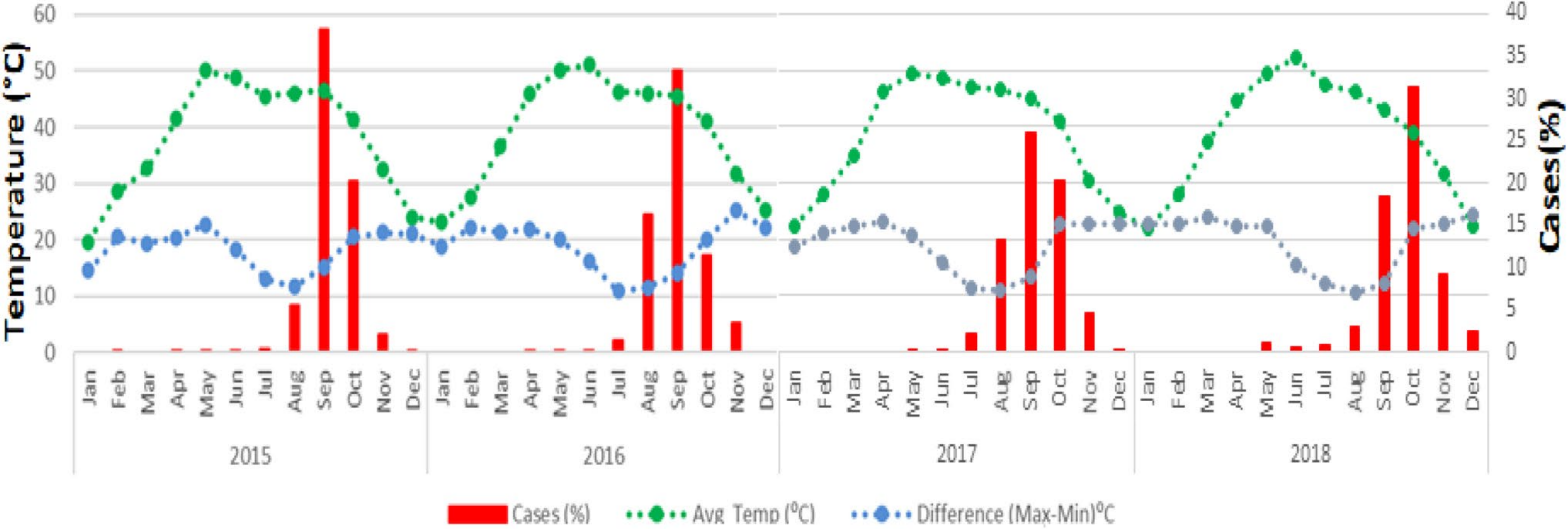
Monthly occurrences of dengue fever and average temperature and difference between the maximum and minimum average monthly temperature, 2015–18, Delhi.

However, it was seen that the difference in the min and max temperature showed a reverse pattern with the dengue cases. The dengue cases were showing increasing with decreasing the temperature difference (Fig. 5). The average temperature difference was reported to be between 25 and 35 °C during the peak of dengue cases.

### Environmental correlates

The Spearman correlation between the dengue cases and climatic variable (Table 2) reveals that the temperature, rainfall and relative humidity were significantly correlated. The correlation of dengue with maximum temperature was significant at lag 1 to 3 months, but it was comparatively high at lag 2 and 3 (0.695 and 0.694 at *p* < 0.01). The minimum temperature was significantly correlated through lag 0 to 3 months with a strong correlation at lag 2 (0.887; *p* < 0.001) and the strength of correlation with minimum temperature was higher compared to maximum temperature. There was also a significant correlation between average temperature and cases of dengue at lag 1 to 3 months, and it was high at lag 2 (0.795, *p* < 0.01). A significant correlation was also seen between the difference in monthly average minimum and maximum temperature at lag 1 and 2 (− 0.675, *p* < 0.01 and − 0.663, *p* < 0.01). The humidity was found significantly correlated, but it was low at lag 0 (0.299, *p* < 0.05). The total rainfall was also significantly correlated with dengue cases through lag 1 to 3 months with the strongest correlation at lag 2 (0.738, *p* < 0.01). However, the cumulative rainfall had the highest correlation at lag 0 (0.795, *p* < 0.01).

**Table 2:** Spearman’s rank correlations of environmental variables with dengue cases.

Predictor variables	#Lag 0	#Lag 1	#Lag 2	#Lag 3
Average max temperature	0.285*	0.553**	0.695**	0.694**
Average min temperature	0.418**	0.761**	0.887**	0.798**
Average temperature	0.331*	0.663**	0.795**	0.789**
Difference temperature (max–min)	− 0.350*	− 0.675**	− 0.663**	− 0.491**
Relative humidity	0.299*	0.260	0.097	− 0.142
Total rainfall	0.234	0.640**	0.738**	0.643**
Cumulative rainfall	0.795**	0.475**	0.115	− 0.283

* < 0.05, ** < 0.01, #Lag time (in months) of environmental variables and reported dengue cases in the current month.

### Probabilistic model

The number of dengue cases increased in the post-monsoon period, indicating a correlation between dengue infection and climatic factors (rainfall, temperature, and relative humidity), as well as providing a basis for a possible empirical model of dengue. The generalized linear model (GLM) using negative binomial regression was used and the findings of the best-fitted model are presented in Table 3. The deviance test (33.220) and omnibus test (likelihood ratio, chi-square 232.832) show that the model was the best fit with response variables of dengue cases and a set of environmental variables as predictors. The criteria of mean deviance (− 2LL/df) close to one and lowest value AIC also confirmed the selected model is the best one. The best fit generalized linear model (GLM) had four predictor variables i.e. the average maximum temperature at lag 2 months, the difference between the maximum and minimum temperatures at lag 1 month, cumulative rainfall (lag 0) and relative humidity (lag 0). All environmental predictors were highly significant under the Wald Chi-square test (*p* < 0.01, AIC = 314.11).

**Table 3:** The generalized linear model using negative binomial regression analysis of the data and the environmental predictors of dengue (2015–2018).

Parameter of model and predictors	Estimate (β)	Std. Error (β)	95% Wald Confidence Interval		Hypothesis Test			Expon-ential (β)	95% Wald Confidence Interval for Exp(β)	
			Lower	Upper	Wald Chi-Square	df	Sig		Lower	Upper
Intercept	4.071	2.0374	.078	8.064	3.993	1	.046	58.615	1.081	3178.63
Temp.Difference (lag1)	− .460	.0717	− .601	− .319	41.093	1	.000	.631	.549	.727
Avg MaxTemp (lag2)	.181	.0558	.071	.290	10.469	1	.001	1.198	1.074	1.336
Cumulative Rainfall(lag0)	.007	.0009	.005	.009	63.993	1	.000	1.007	1.005	1.009
Avg.Relative Humidity (lag0)	− .093	.0188	− .130	− .056	24.329	1	.000	.911	.878	.946

Dependent variable: Dengue cases (number).

Model: Intercept—Constant(β_0_); Predictor variables: Temp Difference(lag1) – a difference of minimum and maximum temperature with lag 1 month; AvgMaxTemp(lag2) – average maximum temperature with lag 2 months; Cumulative Rainfall(lag0) – Cumulative rainfall (January to current month); Avg.Relative Humidity(lag0) – Average relative humidity.

The estimated coefficient of predictors indicates the increase or decrease of the log number of dengue cases with a unit change in its values. The predictors such as the difference in average minimum–maximum temperature at lag 1 and relative humidity had a significant effect on dengue with the coefficient of − 0.460 and − 0.093 respectively. It can be stated that with a one degree Celsius increase in the temperature difference and one % increase in relative humidity, the expected log of dengue cases decreases by 0.460 and 0.093 respectively. Similarly, two months previous maximum temperature with its coefficient of 0.181 shows that with the one-degree increase in maximum temperature, the expected log of dengue cases increases by 0.181. Likewise, it was implied for cumulative rainfall with a coefficient value of 0.007 where one mm increase in cumulative rainfall, the expected log of dengue cases increases by 0.007.

The difference in average maximum and minimum temperature [IRR 0.631, 95% CI 0.549–0.727], maximum temp at lag 2 [IRR 1.198, 95% CI 1.074–1.336], cumulative rainfall [IRR: 1.007, 95% CI 1.005–1.009] and relative humidity [IRR 0.911, 95% CI 0.878–0.946] were significant at *p* < 0.01. The results indicate that maximum temperature and cumulative rainfall had a significant positive impact on dengue incidence while the difference in average maximum and minimum temperature, and relative humidity had a reverse effect on dengue incidence.

**Figure 6:**
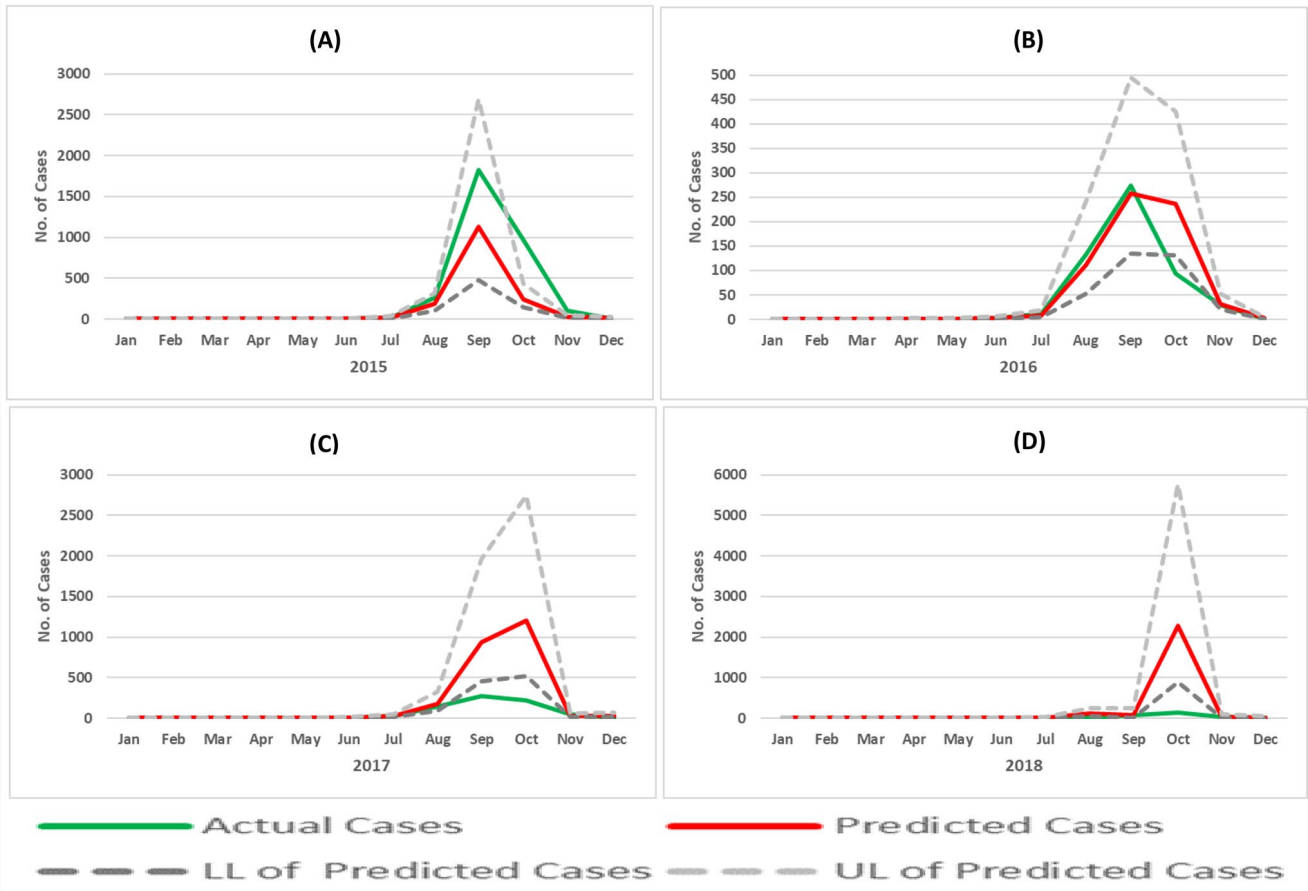
Actual and predicted values of dengue cases with best fit Generalized Linear Model (GLM) using Negative Binomial Regression (2015–2018).

The actual and estimated dengue cases using the model (GLM) with the upper and lower limit of predicted values of mean response are shown in the line graph (Fig. 6). The lines showing the actual and mean estimated dengue cases are very close indicating the best fit for the years 2015 and 2016 represented in Fig. 6A,B respectively. However, the model has underpredicted the cases during the peak period of September and October in 2015. Similarly, the model has over predicted the dengue cases in the years 2017 and 2018 (Fig. 6C,D). The highest predicted dengue cases by the best fit model was 1128 (95% CI 472–2693) in September 2015; 258 (95% CI 134–495) in September 2016, 1199 (95% CI 524–2748) in October 2017; and 2270 (95% CI 894–5768) October 2018. The larger gap between the actual (higher) and predicted values(lower) of dengue cases indicates the dengue epidemic in 2015, whereas it was reversed in 2017 and 2018 showing the epidemic was reverted by health administration due to early action and better management of cases. However, it is important to note that one peak was observed in September in the year 2015 and 2018; but it was shifted towards October showing two visible peaks (September–October) in the years 2016 and 2017.

## Discussion

Even though several studies were performed earlier^1,7–10^, the changes in climatic conditions make it imperative to revisit and re-examine the impact of climate change on the occurrence of dengue cases. The alteration in climate could change both the spatial and temporal dynamics of dengue ecology by increasing vector ranges, broadening the duration of vector activity, and snowballing the mosquito’s infectious period^14^.

The seasonal pattern of dengue cases was similar every year (2015–2018) in Delhi which was also reported by others^10^. Our findings reveal that the dengue cases progressed from July to August, hit the highest point in September to October and declined by December. Dengue cases were recorded high during the post-monsoon period^10^.

Analysis of dengue cases with monthly average max and min temperature revealed that the number of reported cases was the highest from August to October across the study period (2015 to 2018) which was a few months after the highest average max and minimum temperature.

The average maximum temperature(lag 0–3 months) was significantly correlated with dengue cases and it was high at lag 2 and 3 months (0.695 and 0.694; *p* < 0.01) as reported in another study^10^, however, another study reported at lag 0^13^. However, a non-significant effect was also reported in another study^15^. This is because the temperature is an important determinant of egg and immature mosquito development, biting rate, the development time of virus in the mosquito (extrinsic incubation period), and survival at all stages of the mosquito life cycle^1^.

The effect of minimum temperature (lag 0–3) was also significant, but a strong correlation at lag 2 was observed (0.887; *p* < 0.001) as reported by others^13^. The temperature at peak of dengue was between 25 and 27 °C similar to another study that has shown temperatures in the range of 20 to 31.7 °C have provided a suitable environment for breeding and abundance of Aedes mosquito species and thereby increasing the risk of dengue cases^16^.

The correlation between average temperature and dengue cases was significant and high at lag 2 (0.795, *p* < 0.01), while 0–3 months lag was reported in another study^17^. However, a moderate positive correlation was indicated between average monthly temperature and dengue cases^15^ while contrasting results were reported in a study by Su^18^.

A significant correlation was also seen between the difference in monthly average minimum and maximum temperature at lag 1 and 2 with dengue cases (− 0.675 and − 0.663, *p* < 0.01). The negative correlation indicates reverse relation between them. However, the diurnal temperature range (DTR) was reported to be associated with the dengue epidemic^19,20^. High mean temperatures with narrow daily temperature variation, are important for dengue transmission as it influences the biology and vectorial capacity of Ae. Aegypti^21^. It is highly probable that as the number of cold days and nights decreases and the number of warm days and nights increases on the global scale (IPCC), it would impact dengue incidence.

The temporal trend across seasons revealed a rise in the occurrence of dengue fever in the monsoon seasons and post-monsoon seasons. The dengue cases reached the peak following the months with the highest rainfall, post-monsoon (Fig. 1) also reported by another study in Delhi^10^ which may be related to inherent delays between weather conditions and their impact on mosquito populations, virus replication with their subsequent influence on transmission patterns^3^. However, rainfall was not associated with dengue incidence in the Chitwan district of Nepal^13^.

The total and cumulative total rainfall (lag 1–3 months) were significantly correlated and it was high for total rainfall at lag 2 (0 0.738, *p* < 0.01) and cumulative rainfall lag 0 (0.795, *p* < 0.01) as reported by others^3,10,22^. Rainfall was significantly related to dengue as precipitation provides habitats for the aquatic stages of the mosquito life cycle and strongly influences vector distribution^14^, however, extreme rainfall decreases in dengue risk due to adverse impact on vector habitat^23^.A low correlation between humidity and dengue cases was observed as compared to rainfall as reported by others^3^. The humidity (lag 0) was found significantly correlated with dengue (0.299, *p* < 0.05), however, it was reported at lag 0 and 2^9,13^.

The Generalized linear model (GLM) using negative binomial regression was fitted to know the combined effect of environmental factors. The maximum temperature and cumulative rainfall had a significant positive impact on dengue incidence while the difference in maximum and minimum temperature, and relative humidity had a reverse effect on dengue incidence. Similar results were reported in a study conducted in Delhi and rainfall, temperature, and humidity at lag 2 were the significant predictors of dengue^10^. A study in Nepal indicated that minimum temperature at lag 2, the maximum temperature at lag 0, the maximum temperature at lag 3, and relative humidity at lag0 were significant predictors in the model^13^. In a study in Cambodia, the model reflected average temperature, maximum temperature, minimum temperature and rainfall as significant predictors of dengue^17^, while another study in Dhaka city revealed the best fit for maximum temperature, rainfall and humidity at lag 2 months^8^. With such a prediction model it is possible to have better control measures and preparedness for better case management to avoid the epidemic. However, the best fit model of this study is shown to overestimate the number of dengue cases during peak season for the years 2017 and 2018. The wide gap between actual and predicted may be attributed to various other underlying factors such as the presence of susceptible in the population, adopted effective control measures, better case management, case-reporting, better awareness, etc. which have not been considered in the model.

The correlation between dengue incidence and weather factors also seemingly varies by locality, suggesting that a future dengue early warning system would likely be best applied at a local/regional scale, rather than at a nationwide level. The present study is consistent with findings of other studies^24–26^, that a persistent peak in dengue cases each year following the highest rainfall and temperature, indicated the influence of the preceding month’s climatic factors, which was comparable to other countries^17,27,28^, a time during which the mosquito can develop and contaminate the population. In a study on the dengue situation in India, the high transmission potential was also reported throughout the monsoon period^29^. Other studies in Taiwan, Thailand, Brazil, Singapore, etc. also show the association between dengue incidence and seasonal patterns in temperature, relative humidity, and rainfall^1,7–10^. The time lag can be also explained by the influence of weather conditions on the biological development of the mosquito vector, including prolonged egg hatching periods and the propensity of Aedes eggs to survive without water for many months^27^.

The limitation remains that the dengue data is for the entire Delhi and not area-specific hence dengue cases have been taken for the entire Delhi. Therefore, accessing climate data and dengue cases for Municipalities wise was not possible. More years of data need to be studied further to validate the best fit model. The study needs to be extended to socio-demographic components such as population growth, travel or migration rate, water storage habit, etc. This work has been limited to Delhi only, hence future studies encompassing diverse geographic regions should also be included.

## Conclusion

This study has identified a significant effect of environment variables i.e. the average minimum temperature at lag 2 and cumulative rainfall at lag 0 as potential positive contributors whereas the difference in temperature at lag1 and humidity at lag0 were negative contributors to increasing dengue cases in Delhi. In conclusion, these findings demonstrate that transmission of dengue occurs almost year-round, nevertheless, public health preparedness should be focused on-peak periods to cope with potentially large influxes of patients. The study indicates that effective management of dengue outbreaks, an in-depth understanding of the dynamics of not only virus, host and vector, but also local climatic factors specifically in the context of global climate change is required. It is suggested that the vector control program for dengue containment be implemented from June to July for more effectiveness.

## Data Availability

Data are available with the health administration of Delhi and the Indian Meteorological Department which was provided only for research purposes. However, it can be shared after taking permission of the concerned authority.

## Abbreviations

GLM: Generalized linear model
DF: Dengue fever
DHF: Dengue haemorrhagic fever
DSS: Dengue shock syndrome
IPCC: Intergovernmental panel of climate change
NCT: National capital territory
NBR: Negative binomial regression
AIC: Akaike's information criterion
IRR: Incidence rate ratio
